# Insights into a dual-phase steel microstructure using EBSD and image-processing-based workflow

**DOI:** 10.1107/S1600576722004265

**Published:** 2022-06-01

**Authors:** Maxime Mollens, Stéphane Roux, François Hild, Adrien Guery

**Affiliations:** a EDF R&D, Site des Renardières, 77818 Moret-sur-Loing, France; b Université Paris-Saclay, CentraleSupélec, ENS Paris-Saclay, CNRS, LMPS – Laboratoire de Mécanique Paris-Saclay, 91190 Gif-sur-Yvette, France

**Keywords:** electron backscatter diffraction (EBSD), orientations, duplex stainless steels, segmentation, classification, image analysis, microstructures, crystallographic phases

## Abstract

Quantitative metallography to understand the morphology of different crystallographic phases in a material often rests on the segmentation and classification of electron backscatter diffraction (EBSD) maps. Image analysis offers rich toolboxes to perform such tasks based on ‘scalar’ images. Embracing the entire wealth of information provided by crystallography, operations such as erosion, dilation, interpolation, smoothing and segmentation require generalizations to do justice to the very nature of crystal orientations (*e.g.* preserving properties like frame indifference). The present study gives such extensions based on quaternion representation of crystal orientations. A dual-phase stainless steel specimen is used to illustrate the different steps of such a procedure.

## Abstract 2

In this article, an image-analysis-based method is applied to electron backscatter diffraction (EBSD) data in order to recover specific properties in a complex microstructure, and some new dedicated tools are developed and compared with existing methods.

## 1. Introduction

Understanding microstructures is of key importance to predict mechanical properties in advanced system components. In nuclear power plants, the prediction of the behavior of primary coolant system pipes is mandatory. These components undergo severe thermal and mechanical loadings in their in-service environment (Bethmont *et al.*, 1996 ▸; Jayet-Gendrot *et al.*, 2000 ▸). Long-term microstructural changes are likely to degrade material properties by modifying deformation mechanisms and stress distributions under loadings (Verhaeghe *et al.*, 1997 ▸; Baczmański *et al.*, 2016 ▸; Wang *et al.*, 2016 ▸).

For polycrystalline materials, the size, shape and organization of individual crystals are key to optimizing the macroscopic constitutive laws and, in the present case, to understanding their changes with aging. In such characterizations, electron backscatter diffraction (EBSD) has seen a tremendous surge of interest from the 1990s onwards, with the development of digital cameras allowing for fast acquisitions of diffraction patterns and increased computer power driving scanning electron microscopy (SEM) systems. EBSD offers noteworthy properties, such as complete characterization of each phase, very high resolution images for strain analyses (Wilkinson *et al.*, 2006 ▸; Britton & Hickey, 2018 ▸; Shi *et al.*, 2019 ▸; Wang *et al.*, 2021 ▸), recent and forthcoming promising advances (*e.g.* transmission Kikuchi diffraction; Sneddon *et al.*, 2016 ▸), and ultra-fast characterization (Wang *et al.*, 2021 ▸). Remarkable progress has been achieved in serial sectioning alongside collection and assembly of 3D EBSD data (Groeber *et al.*, 2006 ▸; Burnett *et al.*, 2016 ▸; Guyon *et al.*, 2016 ▸; Echlin *et al.*, 2020 ▸; DeMott *et al.*, 2021 ▸) and dedicated software (Bachmann *et al.*, 2011 ▸; Groeber & Jackson, 2014 ▸). However, handling and interpreting such results may become very complicated in the case of complex microstructures. Besides, such experimental data feed models of growing complexity to account for realistic microstructures (Bugat *et al.*, 1999 ▸; Mcirdi, 2000 ▸; Panicaud *et al.*, 2011 ▸). Most of the time, quantitative parameter extraction requires the processing of images coming from different acquisition techniques where EBSD plays a significant part. With this feature in mind, numerous image-processing toolboxes have been automated and made user friendly (Schneider *et al.*, 2012 ▸).

Image processing based on any scalar information extracted from crystallographic orientations cannot be objective, in the sense that it depends on the chosen reference frame, whereas the frame-indifference property should be a prerequisite to all crystallographic data processing routes. Generalization of necessary tools used in basic image processing for segmenting phases to standard EBSD data is therefore of great interest. Dedicated post-processing procedures have been developed to significantly improve the accuracy of orientation measurements by applying appropriate filters to crystallographic data (Humphreys, 2001 ▸; Chen & Thomson, 2010 ▸; Hielscher *et al.*, 2019 ▸) or directly to EBSD patterns (Godfrey, 2004 ▸; Wright *et al.*, 2015 ▸). The quaternion orientation formalism adopted in many EBSD processing codes is well suited for this development and will be used here along with some plotting functions available in the *MTEX* Matlab toolbox (Bachmann *et al.*, 2010 ▸). Quaternions have been used extensively in monitoring of systems involving orientations (*e.g.* aerospace, robotics) for their computer-friendly encoding of rotations. They have also been used in image processing (Chen *et al.*, 2015 ▸; Xu *et al.*, 2015 ▸) to handle color properties, even though 3D rotations or deformations have no physical meaning in color spaces. For example, it would be irrelevant to encode colors in inverse pole figures, as used for orientations. Hence, quaternions are convenient beyond their physical relevance; but for orientations, the latter aspect allows for the formulation of a distance between orientations that is objective and does not suffer from any singularity (as would be the case with Euler angles).

The present article intends to illustrate a workflow composed of dedicated tools to classify and segment crystal-indexing data on materials with complex microstructure layouts. One emblematic example of such materials is cast dual-phase stainless steel, out of which elbow tubes and connections of pressurized water reactors are made. This material will be used in the following analyses to illustrate the discussion. Its microstructure covers a very broad range of scales with characteristic features ranging from 1–10 µm up to several millimetres. The microstructure is ideally suited for achieving specific strength and corrosion resistance requirements (Solomon & Devine, 1982 ▸; Charles, 2009 ▸; Charles & Chemelle, 2012 ▸). However, this material suffers from thermal aging over the years, and becomes slowly more prone to damage and microcrack initiation, which contribute to an increase of the risk of failure (Bonnet *et al.*, 1990 ▸; Chopra, 1991 ▸; Chung, 1992 ▸; Le Delliou & Saillet, 2015 ▸). The complex spatial organization of austenitic and ferritic phases (crucial for the material properties) offers a great challenge to illustrate orientation processing.

The outline of the article is as follows. First, the material and its peculiar microstructure are introduced in Section 2 to highlight distinctive scales that are relevant for mechanical models. The formalism for handling crystal orientations and associated image-analysis tools based on previous observations are then defined in Section 3. A direct application to the studied material and subsequent morphological analyses are shown in Section 4. Finally, the benefits of the introduced methods on the case of interest are discussed in Section 5, alongside some proposed improvements.

## 2. Material and microstructure

**Figure 1:**
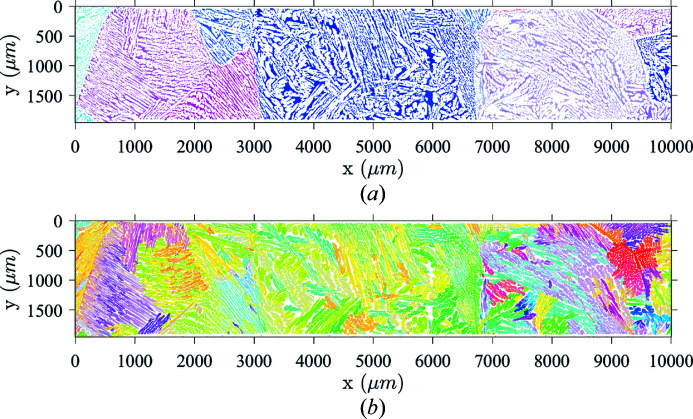
The microstructure of CF8M dual-phase stainless steel. (*a*) Large ferritic grains obtained from primary solidification. Only the α phase is displayed. (*b*) The austenitic lath network growing within ferritic grains.

The material of interest is CF8M alloy, which is a cast dual-phase stainless steel used in the primary loop components of French pressurized water reactors. It consists of two constitutive phases, namely, ferrite and austenite in a 25/75 wt% ratio. Its microstructure exhibits elongated austenite laths of ∼10 to 50 µm width embedded in a ferritic matrix (Fig. 1 ▸).

**Figure 2:**
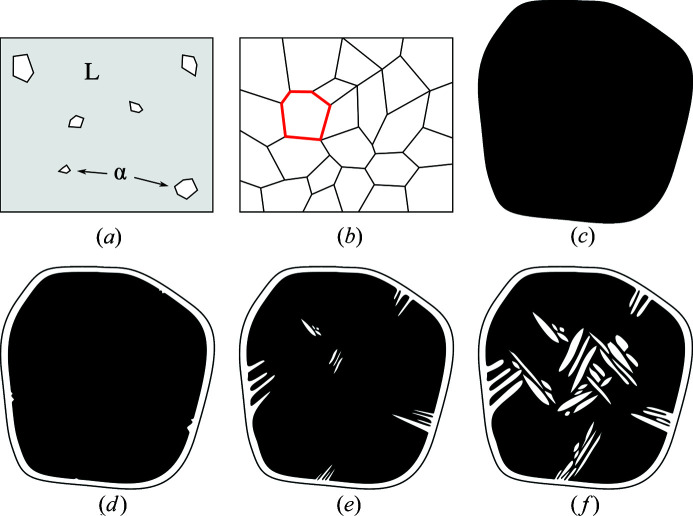
Schematic representation of solidification mechanisms of CF8M duplex stainless steel. The two primary phase transformations occurring during cooling are illustrated. Understanding the physical mechanisms will further help to guide the classification and segmentation methods. (*a*) First phase transformation from liquid to α-ferrite. (*b*) Common polygonal ferrite grain network resulting from full solidification. (*c*) Initial primary ferrite grain taken from (*b*). (*d*) Austenite (in white) starting to grow at ferrite grain boundaries. (*e*) Austenite growth forming laths. (*f*) Ferrite–austenite nested layout.

Looking at the solidification path is instrumental for reading orientation maps of this material and identifying the different scales. A schematic description of the main steps is given in Fig. 2 ▸. The chemical composition is such that solidification starts with a liquid to α phase transformation resulting in a standard assembly of millimetre-size polygonal grains. The polygonal grain shape is easily observed at room temperature [Fig. 2 ▸(*a*)].

A second and quite unusual solid/solid phase transformation from ferrite to austenite takes place further along the cooling process. Two growth configurations have been identified on crystallographic orientation maps for the second phase. First, nucleation is likely to occur where the disordering energy is high (*i.e.* at ferritic grain boundaries and most likely at triple points where three grains meet). Being schematically planar, primary ferritic grain boundaries are replaced by a thin continuous austenite lath (whose thickness is of the order of hundreds of micrometres). Secondary austenite laths grow from these boundaries and into the ferritic grain to form the observed nested layout. Austenite laths give rise to clusters constrained by variant selection mechanisms (Kundu *et al.*, 2012 ▸; De Jeer *et al.*, 2017 ▸; DeMott *et al.*, 2021 ▸) resulting in preferential morphological and crystallographic parameters inside a cluster. Groups of austenite laths with similar orientation are easily observed [Fig. 2 ▸(*b*)].

The macroscopic mechanical modeling, which is a motivation for such study, leads to identifying four scales in this microstructure. The first (smallest) scale is the mono-crystalline austenite lath with constant crystallographic orientation (or the ferritic interlayer). The second scale with meaningful mechanical parameters is associated with the austenite lath clusters characterized by constrained morphological and crystallographic parameters. The third scale is delimited by the primary ferritic grain layout in which clusters are nested. The fourth mechanical scale is the representative volume element of ferritic grains. The aim of multiscale models is to retrieve spatial boundaries of each intermediate scale and endow them with appropriate mechanical features. The smallest scale is easy to delineate. The larger scale is a theoretical extension of the layout given by primary ferritic grains. Hence, only the two intermediate scales have undetermined boundaries. In the following, it is proposed to retrieve these boundaries by considering crystal orientations.

## 3. Classification of EBSD data

Phase identification from optical or SEM images in metals has been a major task for a long time (Tamura *et al.*, 1978 ▸; Fischmeister, 1981 ▸; Osmont *et al.*, 2000 ▸), and still benefits from the latest advances in computer power and classification algorithms (Ducato *et al.*, 2013 ▸; Azimi *et al.*, 2018 ▸) to exploit the relationships between quantitative phase morphology and mechanical behavior. Such images are acquired from a wide range of imaging systems including EBSD. However, scalar fields of such images do not integrate crystal symmetries and consequently do not do justice to the physical meaning of crystallographic orientations. Hence, suited extensions of classification and segmentation operators in the crystallographic orientation space need to be introduced.

Similarly, grain-boundary identification using indexed EBSD data has been the focus of substantial efforts (Groeber *et al.*, 2006 ▸; Bachmann *et al.*, 2011 ▸; McMahon *et al.*, 2013 ▸). More advanced processing of crystallographic orientations may also give access to underlying properties of complex microstructures. For instance, parent-grain reconstruction has been a major focus in recent years (Cayron *et al.*, 2006 ▸; Germain *et al.*, 2012 ▸; Wang *et al.*, 2019 ▸). Between these two cases lie microstructures for which morphological parameters are visible but require some processing to be properly quantified. In the case of CF8M, each phase transformation leaves notable features guiding the orientation map processing (Fig. 2 ▸). For example, spatial boundaries of primary ferritic grains are easily identifiable by separating phases and focusing on ferrite in the spatial orientation maps. Regarding austenite, smooth orientation gradients seem to be correlated with the evolution of morphological properties.

In light of the microstructure shown in Fig. 1 ▸, suitable tools must be developed. The two phase orientation maps, colored using a standard inverse-pole-figure coding, unveil the aforementioned microstructure scales. Large EBSD maps only include a small number of ferritic grains owing to their millimetre sizes. Hence, ferrite orientations correspond to a few distinct clusters randomly distributed in the orientation space. Austenite orientation maps are much more complex and exhibit clusters with homogeneous or continuously varying crystallographic orientations. Their representation in the orientation space results in large and diffuse clouds of scattered points. At the intermediate scale, each lath cluster corresponds to a similar orientation (and thus, in the orientation space, the corresponding points are grouped into a narrow region), while distinct lath clusters appear as distant sets of points in the orientation space. Thus, the boundaries of these clusters will be retrieved by processing ferrite and austenite maps hierarchically in compliance with the above-described nested microstructure layout.

When considering a unique phase, its orientation maps contain a large fraction of missing data (which correspond to the other phase). Processing is thus difficult as it has to accommodate this missing information. Thus some kind of ‘inpainting’ will be needed, and it is important that such inpainting does not misrepresent the actual microstructure and preserves frame indifference [inpainting a rotated map (with an arbitrary rotation) or rotating the inpainted map should be equivalent]. With this aim, a dilation operation is introduced. Furthermore, classification and segmentation of orientations are needed. Here, ideas inspired by standard methodologies such as *K*-means (Hartigan & Wong, 1979 ▸) or ‘connected-component’ algorithms (Di Stefano & Bulgarelli, 1999 ▸) will be used. However, for these different processing operations (dilation, *K*-means, connected components), one must properly extend their formulation to account for the fact that, rather than ‘gray levels’ (*i.e.* scalars), crystal orientations are to be considered, and hence a fair definition of distance between two orientations, for instance, is essential. It is the purpose of the following sections to provide such an extension.

All orientation data used in this article were acquired on a TESCAN Mira3 600 scanning electron microscope with a 3 µm pixel size (in accordance with the large microstructure) at a working distance of 17 mm and an acceleration voltage of 30 kV.

### 3.1. Representation of crystal orientations

A rotation in the 3D space is conveniently represented by a unit vector 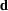 giving the rotation axis and an angle θ. Encoding rotations into unit quaternions, 

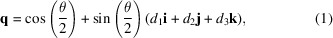

where 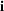 , 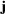 and 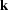 are the basic quaternions, offers a very powerful formalism to handle rotations. This formulation facilitates their composition, interpolation and averaging. Also, it helps in considering symmetries in an elegant way (Frank, 1988 ▸; Morawiec, 2004 ▸).

The ‘distance’ d(...) between two quaternions 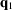 and 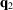 is defined as the angle of rotation 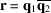 (where the overline denotes conjugation) transforming 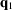 into 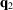 . The cosine of the half-angle of rotation of 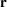 is provided from its real part 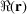 , 

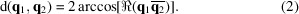

When dealing with crystal orientations, it is essential to account for their symmetries. Again, quaternions offer a simple solution to select as a crystallographic orientation 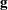 , associated with the quaternion 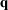 , its representative in the ‘fundamental region’ (Frank, 1988 ▸). Considering all transformations of the crystal symmetry class 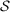 , 

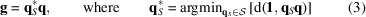

and 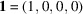 denotes the unit quaternion.

In turn, the distance d(., .) between quaternions is easily extended to define a distance 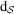 between orientations. Accordingly, the distance between two orientations, 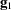 and 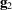 , is the minimum magnitude of the disorientation angle between them, after due consideration of their symmetry,

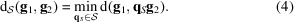

Quaternion mapping of orientations also allows easy interpolation. Using linear interpolation between two quaternions, the average orientation between 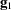 and 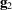 with weights ω and (1 − ω), respectively, reads 

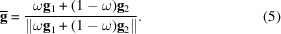

Last, encoding orientations into an axis–angle representation provides a consistent space for representing orientations and misorientations (Frank, 1988 ▸; Krakow *et al.*, 2017 ▸), and allows one to understand how they are distributed in this space. With the aim of keeping consistency with the quaternion formalism introduced herein, orientation 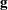 will be represented as a 3D vector, 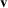 , as given by the imaginary part of the associated unit quaternion 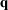 , 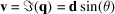 .

### 3.2. Dilation

The EBSD technique provides a map of 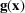 for all analyzed pixels 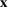 in the region of interest. It is convenient to decompose this map into elementary ones, 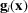 , restricted to a single phase *i* (here ferrite and austenite).

Identifying orientation clusters that are meaningful in the spatial maps requires one to fill empty regions (*i.e.* occupied by the complementary phase) without changing the orientation distribution. To retrieve the transformed parent-phase orientations, one can make use of the resulting child-phase properties or extrapolate the residual parent-phase map. The first method, often referred to as parent-grain reconstruction (Cayron *et al.*, 2006 ▸; Germain *et al.*, 2012 ▸; Wang *et al.*, 2019 ▸), assumes a unique orientation relationship between parent and child phases, and tries to optimally recover the parent orientations through a dedicated algorithm. Since the only required inputs for the method are the parent and child phases, symmetry groups, and an *a priori* guess of the orientation relationship between the two crystallographic frames, the reconstruction can be carried out even when the second phase transformation is complete. Despite its common designation, the method may also be applied to compute a child-phase orientation that would result from transforming the remaining small ferrite islets.

**Figure 3:**
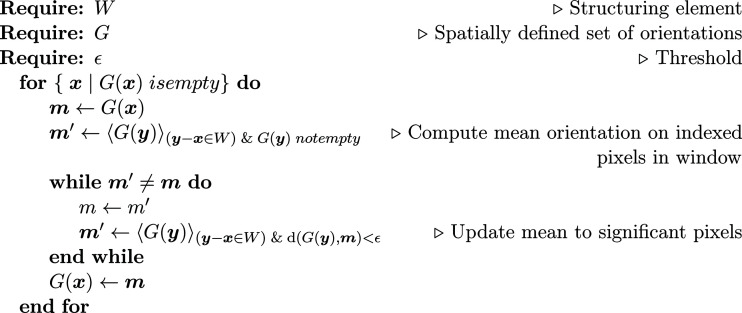
Algorithm 1: orientation map dilation algorithm.

The approach selected in this article is to make direct use of the crystallographic data given by the residual parent phase. Then, the only assumption is a certain amount of regularity in the parent-phase distribution (the shape of grains) and a close-to-uniform orientation distribution inside a single grain. Recovering a full map is achieved using a dilation operator assigning the mean orientation of the neighborhood to empty pixels, which is applied incrementally until all empty pixels are filled (Fig. 3 ▸). The neighborhood is defined by a prescribed structuring element *W* (taken as a 3 × 3 cross shape in the present algorithm). In order to preserve high-magnitude orientation gradients, pixels in the window *W* that are too far from the mean (according to a chosen threshold) are set aside and the mean is computed again. The higher the threshold level, the more boundaries, which are to be highlighted by the process, will be smoothed. Conversely, a threshold-meeting EBSD angular resolution would limit the number of pixels taken into the average and thus ‘sharpen’ the filled pixels. In the following, a threshold of 2.5° was chosen because it was larger than the small orientation gradients that were observed in some austenitic laths, and thus no boundary smoothing was expected to occur.

### 3.3. Erosion

As is done with scalar images, an erosion operator is defined in the orientation space to make small structures disappear and separate larger ones. Typical structures of grains are spherical or elongated clouds in the orientation space, and irrelevant data are isolated points. Hence, a consistent binary image *A* of the orientation space where 0 corresponds to an empty cell and 1 to a non-empty cell is introduced. A regular grid is instrumental to perform the morphological erosion. The eroded data set *G*_e_ is defined from a given data set *G* as 

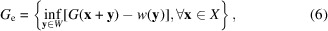

where *X* is the set of coordinates in the rotation space corresponding to *G* and *W* is a subspace where 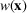 is defined. The grid should be defined according to the quaternion distance metric to avoid distortions. In this framework, the neighborhood is controlled by the grid cell size.

### 3.4. Connected components

For the studied material, each phase orientation has a specific layout in the orientation space, which is preserved by the previously introduced dilation operator. Appropriate tools are to be introduced in order to group orientations sharing similar properties using suitable criteria. According to solidification mechanisms, ferritic grains are scarce, even in large EBSD maps (as their size is millimetric), and correspond to scattered and distinct dense clusters. Hence, an algorithm that can efficiently identify a small number of distinct (and significant) point clouds was used.

A connected-component algorithm was selected. It is a simple clustering algorithm assigning a label to each group of adjacent elementary cells in a given space, where cells correspond to a space discretization. Different spaces may be considered, but because a very similar orientation characterizes each ferritic grain, cells correspond to a discretization of the orientation space. Connectivity of a group of cells is controlled by the cell size. From adjacent pixels in a unique ferritic grain, one may study the distribution of distance, d, of their respective orientations. The standard deviation of this distribution was chosen as half the cell size. The advantage of this procedure is that it is not necessary to set the number of components arbitrarily beforehand.

For data sets including a significant number of grains, it is necessary to add a contribution of the distance in the specimen physical space (*i.e.* to differentiate two distinct grains having close crystallographic orientation). This step is, for example, achieved by segmenting the label map obtained from the connected-component algorithm in the real space.

### 3.5. Gradient

It is necessary to rely on the physical meaning of quaternions to establish a meaningful gradient operator for orientations. The latter should rest on crystallographic rotation angles to highlight grain and sub-grain boundaries, smooth transitions occurring in a grain to accommodate local constraints, and spurious isolated orientation variations due to badly indexed pixels. Accordingly, the previously introduced crystallographic distance becomes a natural descriptor of a crystallographic variation ‘intensity’, and a significant gradient operator is defined as 

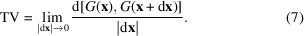

This is translated into a finite-difference version for crystallographic orientation maps,

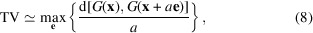

where 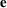 is a unit vector in the 2D plane.

The determination of crystal orientations from acquired Kikuchi patterns can be locally troublesome. Thus, raw indexed data suffer from badly or not indexed pixels. To maximize the classification algorithm’s efficiency, denoising of orientation data with the appropriate filters is helpful. For this, the introduced total-variation operator can be derived in relevant filters. Interesting tools have already been published and implemented in *MTEX* (Steidl *et al.*, 2016 ▸; Hielscher *et al.*, 2019 ▸), *e.g.* a half-quadratic filter that keeps consistency in grain boundaries and strain-induced gradients.

### 3.6. *K*-means classification

**Figure 4:**
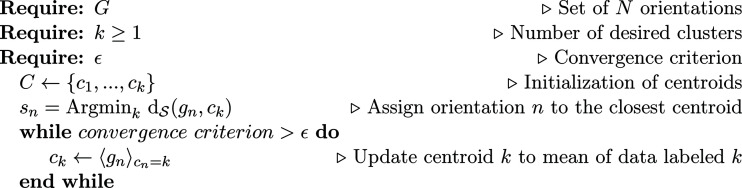
Algorithm 2: *K*-means for crystallographic orientations.

Austenite orientations require one to process large elongated clusters. Thus, a clustering algorithm that is able to separate data into distinct clusters based on statistics is needed. In the present case, it was chosen to rely first on a centroid-based algorithm, namely *K*-means, because it efficiently partitions large data sets (Hartigan & Wong, 1979 ▸) and is therefore instrumental for understanding uncommon γ-phase orientation distributions. The *K*-means algorithm (Fig. 4 ▸) assumes a known cluster number. From an initial guess of the cluster centroid positions, their coordinates are updated to reduce the variance of all data in a cluster until stationarity is reached. By making direct use of the crystallographic orientation metric, point clouds spread near a boundary of the fundamental region are fully recovered by taking crystal symmetries into account. The main drawback is that this algorithm is sensitive to noise and strongly depends on the number of clusters and their initialization. Yet, it constitutes a powerful tool for manually scanning relevant data to be extracted from the full data set. In particular, *K*-means outputs are later used for a specific segmentation detailed in Section 4.1.

## 4. Microstructure analysis through processing of EBSD maps

### 4.1. Processing ferrite orientation

**Figure 5:**
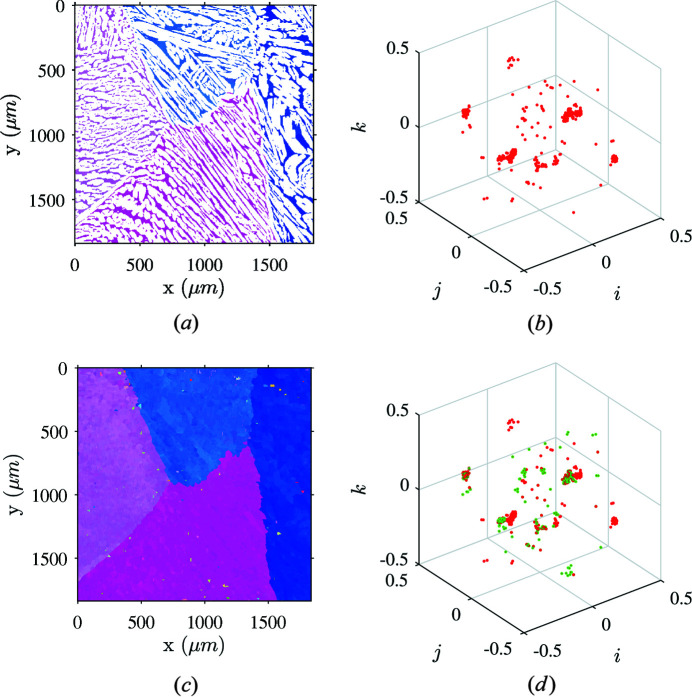
Illustration of the ferrite orientation map dilation process. (*a*) Measured ferrite orientation map. (*b*) Corresponding orientations plotted in the orientation space. (*c*) Dilated ferrite orientation map revealing the boundaries of the primary ferrite grains. (*d*) Dilated map orientations plotted in the orientation space. Green points refer to empty pixels on the initial map that are filled by the dilation process.

Splitting the two phase maps favors a clearer reading of their layout and unveils the three smaller scales but leaves some locations empty. As intermediate-scale boundaries are identified through distinct crystallographic gradients, empty pixels of each map must be filled as seamlessly as possible. For this purpose, an iterative nonlinear average ‘inpainting’ procedure was developed. An empty location gets assigned the mean orientation of its neighborhood ignoring empty pixels and orientations whose distance is too far from the mean according to a prescribed threshold as discussed above. In the case of CF8M steel, applying this procedure to the ferrite orientation map (Fig. 5 ▸) highlights primary α-grain boundaries. Although ferrite represents only ∼25% of the map, 75% must be filled with the procedure, thus giving a significant weight to badly indexed pixels. Consequently, some noise originating from automated EBSD indexing gets amplified by the algorithm [as highlighted by green points in Fig. 5 ▸(*d*)] but the grains remain identifiable.

**Figure 6:**
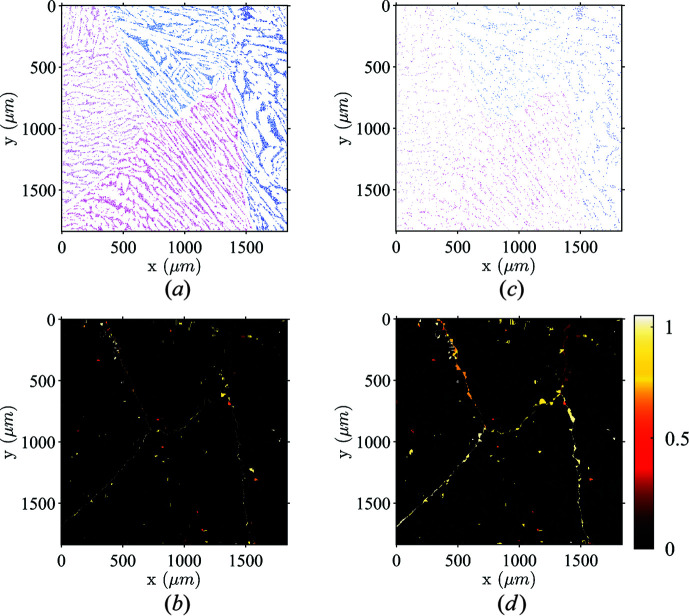
Influence of the initial spatial distribution of correctly indexed pixels on the dilation process. In both test cases, a random selection of ferritic grain pixels was removed, namely, 60% in case (*a*) and 95% in case (*b*). The angular distance of orientation (in degrees) between the dilated map obtained from the full set of ferritic pixels and the depleted cases is shown in (*c*) and (*d*), for 60 and 95% depletion, respectively.

A similar dilation algorithm was implemented and tested by Wright (2006 ▸) to replace some badly indexed pixels. The major difference with the proposed algorithm lies in the orientation attributed to bad pixels. Instead of an average operator, the bad pixels are assigned the orientation of a neighboring pixel until all pixels belong to a grain. The neighboring pixel is chosen using a minimum grain size and a confidence index (Field, 1997 ▸) criterion. Results obtained by Wright underline the significant influence of the initial spatial distribution of reference pixels (*i.e.* pixels that are correctly indexed in the phase of interest). To test the robustness of the proposed dilation step, simulated ‘scarce’ data were extracted from the presented ferrite orientation map. In Figs. 6 ▸(*a*) and 6 ▸(*b*), ferrite pixels were removed at random with a proportion of 60 and 90%, respectively. The dilation step recovers most of the ferritic phase, as judged from the angle distance between the dilated maps starting from the entire set of ferritic pixels and the randomly depleted cases shown in Figs. 6 ▸(*c*) and 6 ▸(*d*). Grain boundaries are more fragile, and are observed in those differences, but the most striking feature is the robustness of the procedure.

### 4.2. Generation of parent phase from child phase

Parent-grain reconstruction algorithms have been extensively developed recently. In the following, the method proposed by Niessen *et al.* (2022 ▸) and implemented in *MTEX* was used. The algorithm starts from an initial guess of the orientation relationship between the two phases and then optimizes it iteratively. Austenite orientations are then clustered into the number of possible child variants given by the computed relationship, and the local parent orientations are determined through a voting scheme. As an initial guess, the Pitsch orientation relationship (Pitsch, 1959 ▸) gave the best fit at convergence and was therefore used to reconstruct the ferrite orientation map of the CF8M alloy.

**Figure 7:**
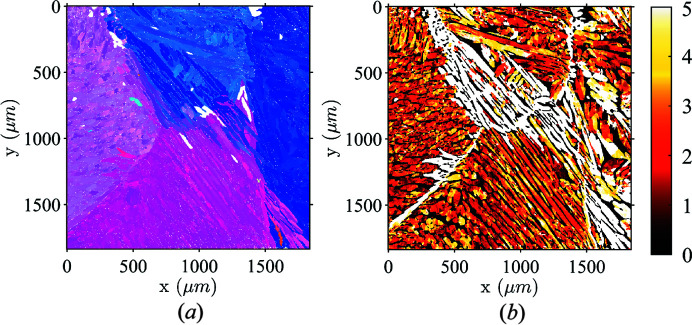
Comparison between recovered ferrite maps using dilation and parent-grain reconstruction methods. (*a*) Ferrite orientation map computed with the parent-grain reconstruction method described by Niessen *et al.* (2022 ▸). (*b*) Angle (in degrees) with the dilated map of Fig. 5 ▸(*c*).

Fig. 7 ▸ shows the reconstructed map of ferrite as provided by the parent-grain reconstruction algorithm [Fig. 7 ▸(*a*)], and the crystallographic distance [Fig. 7 ▸(*b*)] from the raw dilated map of Fig. 5 ▸(*c*). A majority of points (*i.e.* 97%) belonged to a child variant after reconstruction. The two ferrite maps are very close to each other, showing the high quality of the proposed algorithm and the relevance of the Pitsch orientation relationship. The areas where the orientation difference shown in Fig. 7 ▸(*b*) exceeded 5° correspond to prior ferrite grain boundaries, and where, very early on in the cooling process, thin and continuous austenite linings nucleated.

The difficulty for the present algorithm to detect the boundaries correctly is presumably explained by the exceptional propagation of the austenite phase in this material. Not only did austenite appear along ferrite grain boundaries, but it also propagated on both sides of this boundary, keeping a similar orientation. This feature means that the orientation relationship cannot be obeyed in both ferritic grains, and hence on one side the Pitsch relationships had to be violated. This effect prevents the reconstruction algorithm from properly identifying the faulty ferritic grain. By continuity, it inherited the neighboring grain’s orientation. Inside the parent grains where a good agreement with the computed orientation relationship was found, the difference with dilation was rational and stayed below 5°. The precise accommodation of the phases during the casting process still remains unclear, so no conclusion was drawn on whether small changes of the austenite orientation could be attributed to a transmitted small rotation in the prior ferrite phase or to a rotation of the child phase occurring at a later stage. In this regard, the differences between the two processes inside the parent grains, albeit small, may be an interesting observation deserving further investigation.

**Figure 8:**
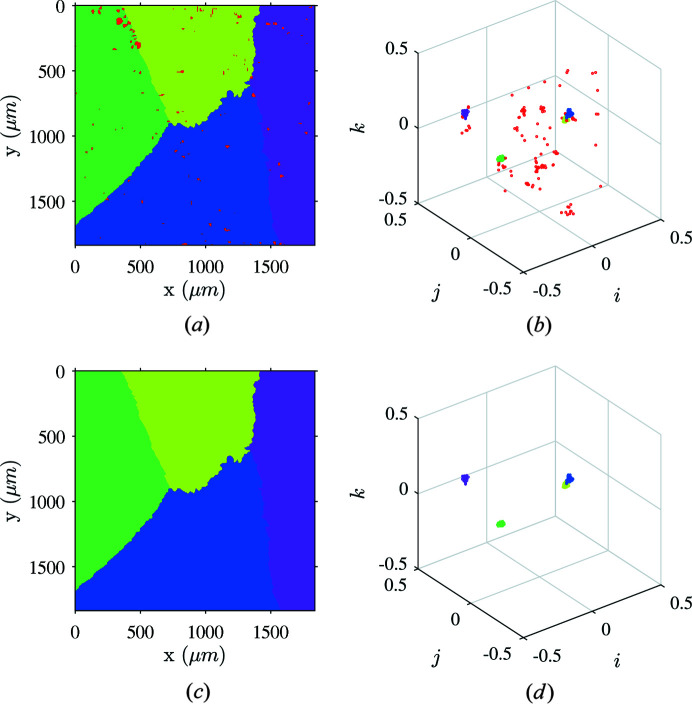
Illustration of ferrite orientation clustering using the connected-components algorithm. (*a*) Labels attributed using the connected-components algorithm in the orientation space. (*b*) Orientations colored according to cluster labels in the orientation space. (*c*) Label map after inpainting. (*d*) Dilated ferrite orientations after inpainting.

Several approaches may be considered to detect isolated clusters considering local orientation density to overcome the influence of outlying points and noise. Density-based clustering using the DBSCAN algorithm was shown to provide satisfactory results for relatively small sets of orientations (Johnstone *et al.*, 2020 ▸). In the present case, large EBSD sets (up to four million points) prevent computing the crystallographic distance matrix for all orientations in a decent computation time. The connected-components algorithm is well suited for such a configuration because checking if adjacent cells are connected is straightforward. The computation time is drastically reduced to a very small cost when compared with DBSCAN. However, orientation clusters lying on an edge of the fundamental zone will be cut if points spread apart from this boundary. To assign equal weight to each sub-area and avoid metric distortions, the grid must be uniform. For the sake of simplicity, a standard voxel was chosen, although it is known that it is not the most appropriate choice (Yershova *et al.*, 2010 ▸) (the earlier introduced distance between voxels is not strictly uniform). Hence, a single parameter (the voxel size) is required. However, the angular distances between orientations considered herein are assumed to be much greater than the heterogeneity of the inter-voxel distance. The labeled clusters identified with the connected-components algorithm are depicted in Figs. 8 ▸(*a*) and 8 ▸(*b*). Cells containing less than 30 orientations are associated with noise and assigned to label 0. An additional cleaning step is performed by deleting those bad pixels and running the dilation process again [Figs. 8 ▸(*c*) and 8 ▸(*d*)].

### 4.3. Processing austenite orientation

**Figure 9:**
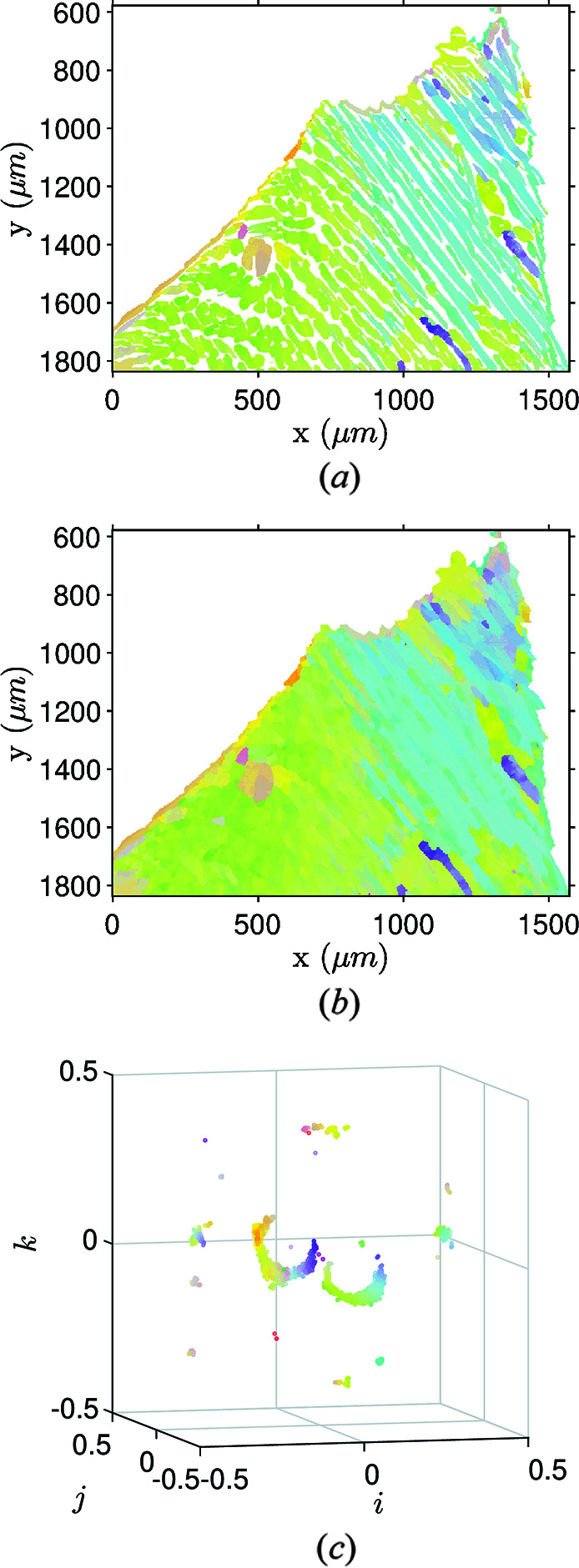
Illustration of the austenite orientation map dilation process in a single ferritic grain. (*a*) Measured orientation map. (*b*) Dilated orientation map. (*c*) Dilated orientations plotted in the ferrite crystallographic reference frame.

It is meaningless to classify austenite orientations on a full EBSD map including several ferritic grains. Crystallographic orientation distributions of the γ phase are strongly related to the primary α grain from which it originated. This feature is highlighted by plotting the child orientations relative to the parent-grain crystallographic frame (*i.e.* plotting the misorientation 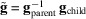 in the orientation space). An example is given in Fig. 9 ▸ corresponding to the ferritic grain attributed to the purple label in Fig. 5 ▸. Each point was colored using a default inverse-pole-figure color map to highlight the γ-phase features. Its orientations are gathered in so-called Bain groups but showcase continuous transitions between commonly reported orientation relationships (*e.g.* Kurdjumov–Sachs, Nishiyama–Wassermann and Pitsch). These Bain groups correspond to the union of symmetrically equivalent orientation relationship representatives (*i.e.* variants) that are close to one another. The continuous aspect manifests itself through smooth crystallographic gradients between variants belonging to a Bain group and morphological gradients on the orientation maps.

**Figure 10:**
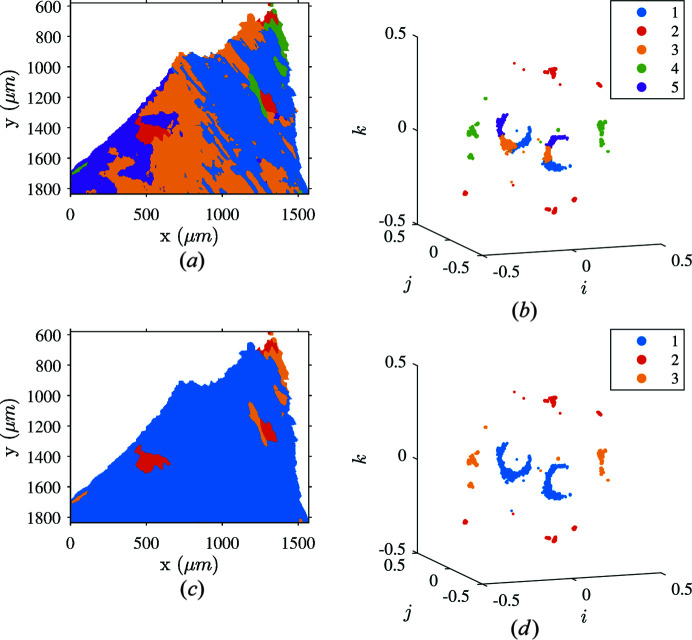
Clustering of austenite crystallographic orientations in a single ferritic grain using *K*-means with *k* = 5 before and after merging adjacent groups. (*a*) Computed clusters illustrated on an orientation map. (*b*) The same clusters in the orientation space. (*c*) Retained classification after merging adjacent clusters. (*d*) Merged clusters represented in the orientation space.

There is an intrinsic issue in reading quaternion plots in this case because orientation clusters are not distinct but rather large and elongated. Thus, a *K*-means tool was used to automatically search for centroids and significant orientation groups in the map. The clustering algorithm was helpful in selecting points that illustrate the continuous aspect of the orientation spread. Clusters identified in the data set of Fig. 9 ▸ are given in Fig. 10 ▸ for five centroids randomly initialized inside the cubic fundamental region. This segmentation clearly depicts the majority of orientations spreading around one Bain group [orientations gathered in clusters 1, 3 and 5 of Figs. 10 ▸(*a*) and 10 ▸(*b*)]. The few remaining points are either on the other two Bain groups (far from usual orientation relationships) or badly indexed [clusters 2 and 4 of Figs. 10 ▸(*a*) and 10 ▸(*b*)]. The segmentation also highlights the continuity between the three major clusters. Clusters 1, 3 and 5 seem to be connected as represented in Fig. 10 ▸(*b*).

The continuous path between different orientation relationships has been highlighted before in face-centered cubic to body-centered cubic (f.c.c. to b.c.c.) transformation paths (Cayron *et al.*, 2010 ▸; Cayron, 2020 ▸), and in the reverse transformation (Stanford & Bate, 2005 ▸; De Jeer *et al.*, 2017 ▸) occurring in the present material. The continuous aspect is strongly believed to be explained by the accommodation of elastic strains in the parent matrix during the α → γ phase transformation (Cayron, 2013 ▸) since continuous orientation-relationship variation accompanies continuous morphological features. The studied dual-phase steel shows a progressive change in orientation over long characteristic lengths as compared with primary ferrite grain sizes, which is an original property. Thus, this feature is likely to influence the local mechanical behavior and should be preserved in the segmentation step. However, strict segmentation of the presented data proved to be inadequate for describing this b.c.c. to f.c.c. transformation feature.

The consistency in the orientation groups computed by *K*-means was recovered by measuring the smallest distance between all pairs of points in different groups. One way around computing the full distance matrix between points in two groups, 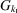 and 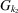 , is to evaluate the distance matrix for a single orientation, 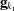 , and to find the orientation in the second group corresponding to 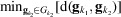 . Carrying out the computation this way, and the other from 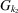 to 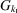 , iteratively allows for the determination of the closest points in the two groups in a very small number of steps. If the distance between the two closest orientations is less than a given threshold, the two groups are merged; otherwise they correspond to distinct orientation clusters.

Figs. 10 ▸(*c*) and 10 ▸(*d*) depict the application of the merging algorithm on the spatial map and in the orientation space. Three clusters remained after merging clusters 1, 3 and 5 of Figs. 10 ▸(*a*) and 10 ▸(*b*) into a unique one labeled 1 in Figs. 10 ▸(*c*) and 10 ▸(*d*). These three clusters roughly correspond to the three Bain groups where continuity in the orientation spread is now preserved. According to Fig. 10 ▸(*c*), the majority of austenite orientations belong to a single Bain group but exhibit a significant orientation spread greater than 10°.

## 5. Conclusion

The use of an image-processing-inspired workflow accounting for crystal-orientation data proved to be useful in reading the complex microstructure of a CF8M dual-phase steel. The chosen hierarchical approach using different classification and segmentation steps emphasized different specific features of the studied microstructure. The parent ferritic grain structure was fully retrieved from combined dilation–erosion and classification using a connected-components algorithm. The structure of austenitic laths was determined from similar techniques. However, the *K*-means algorithm intended for clustering orientations connected to distinct orientation relationships with the parent phase turned out to be insufficient. Regrouping the obtained clusters on the basis of their minimum distance allowed for the restoration of continuity in austenite orientations. Assuming that the b.c.c. to f.c.c. phase transformation was predominantly displacive (*i.e.* diffusionless), the precipitation of austenite at former ferritic grain boundaries resulted in high strain energies. A unique relationship that tended to minimize lattice distortions could not be followed in two α grains that were randomly oriented with respect to each other. Induced strains were accommodated while the precipitate moved farther from the boundary during the cooling process, thereby resulting in smooth morphological and crystallographic orientation gradients in the γ phase.

This work has demonstrated the benefit of using classification and segmentation techniques adapted from image-analysis procedures to crystallographic data. The introduced tools may be useful in various cases from manual investigations to fully automated processing of microstructures, giving a better understanding of the studied material. In the present case, the knowledge of the different scales and their properties was needed for a better understanding of the alloy behavior. The relationship between relative phase orientations and lath morphology is one key ingredient to develop a consistent mechanical model. In future work, the recovered domain geometries (*i.e.* primary ferrite grains, lath packets) will be used to identify different constitutive laws using full field measurements together with the analysis of their microstructure. Furthermore, 3D reconstruction will be considered by generalizing the presented algorithms in the third (missing) dimension.
